# Comparison of Effectiveness and Safety between High-Power Short-Duration Ablation and Conventional Ablation for Atrial Fibrillation: A Systematic Review and Meta-Analysis

**DOI:** 10.1155/2022/6013474

**Published:** 2022-08-16

**Authors:** Shuyu Jin, Lu Fu, Junrong Jiang, Xingdong Ye, Huiyi Liu, Yanlin Chen, Sijia Pu, Shulin Wu, Yumei Xue

**Affiliations:** ^1^Department of Cardiology, Guangdong Cardiovascular Institute, Guangdong Provincial People's Hospital, Guangdong Academy of Medical Sciences, Guangzhou 510080, China; ^2^The Second School of Clinical Medicine, Southern Medical University, Guangzhou 510515, China

## Abstract

**Aim:**

We aimed to evaluate the effectiveness and safety between high-power short-duration (HPSD) radiofrequency ablation (RFA) and conventional RFA in patients with atrial fibrillation (AF).

**Methods:**

Studies comparing HPSD and traditional applications in patients undergoing initial catheter ablation for atrial fibrillation from inception through December 2021 were searched on Pubmed, Medline, Cochrane, and Clinicaltrials.gov.

**Results:**

The meta-analysis included seventeen studies with a total of 4934 patients. HPSD group decreased procedure duration (mean difference (MD) −38.28 min, *P* < 0.001), RF duration (MD −20.51 min, *P* < 0.001), fluoroscopy duration (MD −5.19 min, *P* < 0.001), and acute pulmonary vein reconnection (Odds ratio (OR) 0.40, *P* < 0.001), while improving the freedom from atrial arrhythmia at one year (OR 1.48, 95% confidence interval (CI) 1.12–1.94, *P*=0.005) and rates of first-pass isolation (OR 8.92, *P*=0.001). Compared with the conventional group, freedom from atrial arrhythmia at one-year follow-up was higher in the HPSD group without the guidance of AI/LSI (OR 1.66, *P*=0.01) and studies with a power setting of 40–50 W (OR 1.93, *P*=0.002). Nevertheless, the two groups had similar effectiveness with a power setting of 50 W in the HPSD RFA (OR 1.10, *P*=0.52). There was no difference in complications between the two groups (*P*=0.71).

**Conclusion:**

HPSD RFA was associated with shorter procedure duration, higher freedom from atrial arrhythmia, and comparable safety compared to conventional RFA.

## 1. Introduction

Catheter ablation is recommended as an effective therapy for atrial fibrillation (AF) to reduce the risk of stroke, heart failure, and mortality and improve the quality of life [1]. As the cornerstone of AF catheter [2, 3], pulmonary vein isolation (PVI) aims to produce continuous, transmural, and durable lesions around the pulmonary vein. Remarkably, pulmonary vein reconnection (PVR) could be a vital driver of AF recurrence [4, 5]. The High-power short-duration (HPSD) ablation strategy comprises the use of higher RF power (≥40 W) and shorter duration (5–15 s) of each RF energy application, and HPSD applications result in larger lesion diameters and smaller lesion depths compared to conventional (20–35 W, 10–30 s) applications [6]. Recent studies [7–9] demonstrate that HPSD is safe and efficient enough for treating AF which reduces radiofrequency catheter ablation (RFA) and procedure times without increasing major complication rates. Meta-analyses and randomized controlled studies comparing atrial arrhythmia recurrence and rates of PVR between HPSD and conventional RFA settings with or without the guidance of ablation Index (AI) or lesion size index (LSI) are lacking. Therefore, this meta-analysis compares the effectiveness and safety of HPSD and LPLD settings in RFA for AF [10].

## 2. Methods

### 2.1. Search Strategy and Study Selection

This meta-analysis was performed according to the Preferred Reporting Items for Systematic Reviews and Meta-Analysis (PRISMA) guidelines [11] (PRISMA 2009 checklist in Supplementary material online, Table S1). An all-round search was conducted in Pubmed, Medline, Cochrane, and Clinicaltrials.gov from inception through December 2021 by two reviewers (Shuyu Jin and Yumei Xue) independently. The search involved the following keywords: (“Atrial fibrillation” OR AF) AND (“High power” OR “High-power shorter-duration” OR HPSD)．

### 2.2. Inclusion and Exclusion Criteria

The studies included fulfilled the following criteria: (1) cohort study, case-control study, cross-sectional study, or randomized controlled trial (RCT) conducted in patients with age≥18, with paroxysmal and/or persistent AF undergoing initial catheter ablation; (2) comparison between HPSD RFA and conventional RFA; (3) studies must meet the following for each ablation strategy: HPSD settings: Power≥40 W, with ablation duration of 5 to 15 s per site including the posterior wall; conventional settings: Power ≤35 W, duration >10 s for any ablation; (4) reported outcome data including but not lcomparesdure time, freedom from atrial arrhythmia, total complications, redo-ablation procedure; (5) the follow-up duration was at least 6 months.

The exclusion criteria were as follows: (1) conference abstracts, case reports, review articles, meta-analyses, editorials, or nonEnglish articles; (2) an equivocal study design or group allocation.

### 2.3. Data Extraction and Quality Assessment

A standardized data collection form was extracted by two investigators independently to obtain the following data from each study including name of the first author, year of publication, country of origin, study population, inclusion and exclusion criteria, demographic data of participants, and ablation procedure details. Disagreements were arbitrated by a third person in rereview. The original author was contacted by mail for access if the full text could be obtained. For literature in which the same study populations were reported many times or repeatedly published, only one with the most complete data was included. The quality of these studies was evaluated by two investigators (Shuyu Jin and Yumei Xue) using the Newcastle Ottawa scale (NOS) for observational studies [12] and the Cochrane Collaboration tool for assessing risk of bias of randomized controlled studies (RCTs) [13].

### 2.4. Definitions

  HPSD RFA :  ablation power ≥40 W, including the posterior wall, with ablation duration of 5 to 15 s per site.
  Conventional RFA :  ablation power limited to 20–35 W, with a longer ablation duration of 10–30 s per site.
  Procedure time: time from the application of local anesthesia to the withdrawal of all catheters.
  RF time: total time from the first to the last ablation site.
  Fluoroscopy time: total time for fluoroscopy from the start to the end of the procedure.
  First-pass PVI :  rate of complete PVI after first-pass circumferential RF delivery.
  Atrial arrhythmia recurrence: any symptomatic or asymptomatic atrial arrhythmia lasting >30 s after completing the blanking period post ablation.
  Acute PVR :  acute reconnection was assessed at 20–30 minutes post ablation, and adenosine was administered intravenously (dosed to achieve transient heart block) or waiting for 30 minutes following the last RF application to assess PV reconnection, including spontaneous reconnection and dormant conduction.
  Major complication: complications that required any intervention or prolonged hospital stay including pericarditis, complete atrioventricular block, sinus node dysfunction, phrenic nerve palsy, stroke, ptocardial effusion, vascular access issues, steam pop, esophageal lesions, and death.

### 2.5. Statistical Analysis

Binary variables were expressed as odds ratios (ORs) with 95% confidence intervals (CIs). Continuous variables were analyzed using the mean difference (MD) and the corresponding 95% CI was estimated using the inverse-variance method. A two-sided *P*-value ≤0.05 was considered statistically significant. The fixed-effects model and the random-effects model were considered based on the level of heterogeneity. The heterogeneity of studies was evaluated by Cochran's *Q* and the I^2^ statistic. I^2^ lies between 0% and 100% with larger values showing increasing heterogeneity. I^2^ value > 50% was considered high degrees of heterogeneity and the random model was used in the subgroup analysis or sensitivity analysis excluding the trials that potentially biased the results to avoid publication bias, otherwise, a fixed-effects model was used [14, 15]. We performed sensitivity analysis by omitting one study successively to evaluate the impact of the individual studies on the pooled effect size. Publication bias was assessed using funnel plot and Egger's regression tests (*p* < 0.05 was considered significant). In addition to using the STATA 17.0 statistical software (College Station, TX) for funnel plots and Egger's regression tests, the rest of the statistical analyses were performed using the RevMan version 5.3 (Nordic Cochrane Center; The Cochrane Collaboration, 2014).

## 3. Results

### 3.1. Eligible Studies

**Figure 1:**
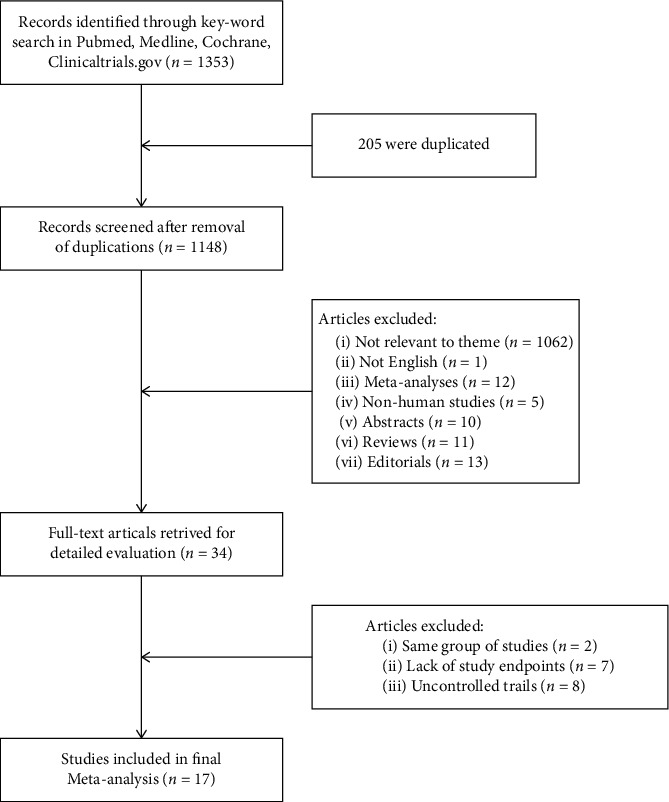
PRISMA flowchart of detailed search progress.

The flowchart of the detailed search progress is illustrated in Figure 1. After removing the duplicated articles and browsing the abstracts, titles, or full texts, consequently, seventeen studies [16–32] with 4934 patients were enrolled in this meta-analysis. Among these studies, ten [16, 18–21, 23, 24, 27–29] were retrospective cohort studies and seven [17, 22, 25, 26, 30–32] were prospective studies in which only one study [30] was a randomized controlled trial (RCT).

### 3.2. Study Characteristics

Baseline characteristics among these studies are shown in Tables 1 and 2. There were 2397 who underwent HPSD RFA and 2537 who underwent conventional ablation procedures. In the HPSD group and conventional group, the mean age was 63.53 and 61.88 years, with 67.58% and 70.40% males, respectively. The baseline characteristics were not a significant difference between the two groups and the followup duration was at least 6 months. Study quality assessed by NOS demonstrated that seven studies scored 9 and ten studies scored 8, which indicated the good quality of the included studies (Table 3).

### 3.3. Primary Pooled Analysis

**Figure 2:**
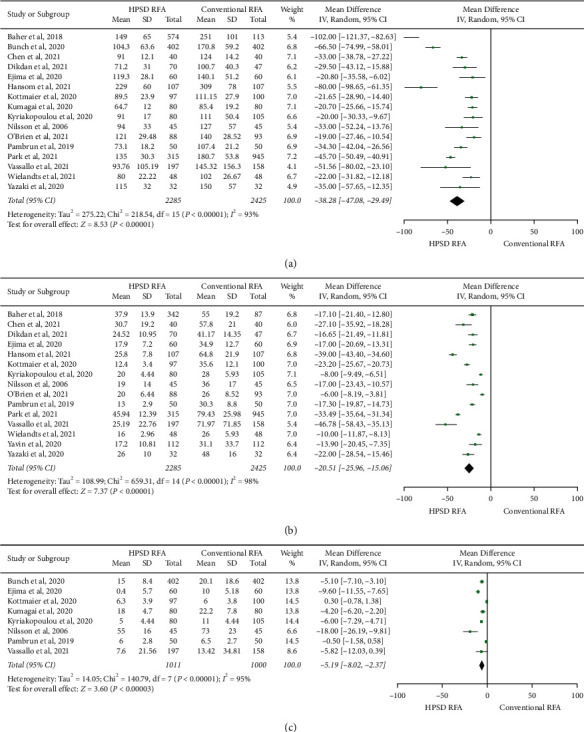
Forest plots of the primary pooled analysis demonstrating the effect of high-power short-duration RFA vs. conventional RFA in patients with atrial fibrillation. Data are mean duration and standard deviation in each group and weighted mean difference. The horizontal line is the 95% CI. The diamond shape is the pooled mean difference of all studies. CI: confidence interval; RFA: radiofrequency ablation. (a) Total procedure duration. (b) Total RF duration. (c) Total fluoroscopy duration.

**Figure 3:**
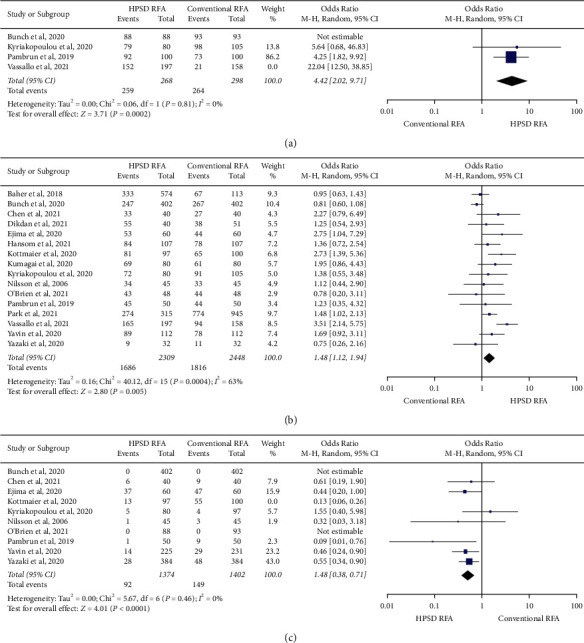
Forest plots of the primary pooled analysis demonstrating the effect of high-power short-duration RFA vs. conventional RFA in patients with atrial fibrillation. Data are events in each group and weighted odds ratios. The horizontal line is the 95% CI. The diamond shape is the pooled mean difference of all studies. CI: confidence interval; RFA: radiofrequency ablation, PVR: pulmonary vein reconnection. (a) First-pass isolation, (b) freedom from atrial arrhythmia at one year, and (c) acute PVR.

**Figure 4:**
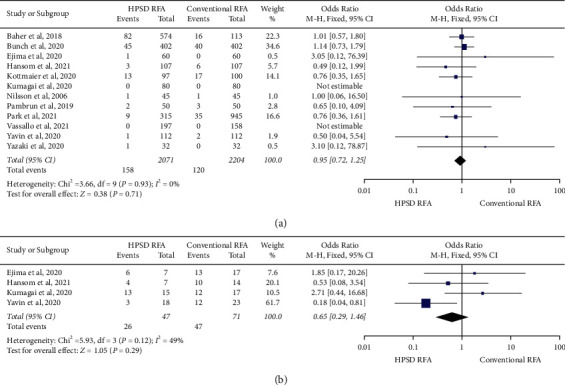
Forest plots of the primary pooled analysis demonstrating the effect of high-power short-duration RFA vs. conventional RFA in patients with atrial fibrillation. Data are events in each group and weighted odds ratios. The horizontal line is the 95% CI. The diamond shape is the pooled mean difference of all studies. CI: confidence interval; RFA: radio frequency ablation; PVR: pulmonary vein reconnection. (a) Total complications. (b) PVR during redo procedures.

Total procedure duration was significantly shorter in the HPSD RFA group compared with the conventional RFA group (MD −38.28 min (95% CI −47.08 to −29.49); *P* < 0.001) (Figure 2(a)). Compared with the conventional RFA group, total RF duration (MD -20.51 min (95% CI −25.96 to −15.06); *P* < 0.001) (Figure 2(b)) and total fluoroscopy duration (MD −5.19 min (95% CI −8.02 to −2.37); *P* < 0.001) (Figure 2(c)) were also significantly shorter in the HPSD RFA group. First-pass isolation (OR 8.92, 95% CI 2.40–33.09, *P*=0.001) (Figure 3) and freedom from atrial arrhythmia at one year (OR 1.48, 95% CI 1.12–1.94, *P*=0.005) (Figure 3) were significantly higher in the HPSD RFA group when compared with the conventional group. Acute PVR was significantly lower in the HPSD RFA group (OR 0.40, 95% CI 0.23–0.69, *P* < 0.001) (Figure 3). There was no difference between the two groups regarding total complications (OR 0.95, 95% CI 0.72–1.25, *P*=0.71) (Figure 4). Among these studies, only four studies described PVR during redo procedures, and there was no difference in PVR between the two groups (OR 0.65, 95% CI 0.29–1.46, *P*=0.29) (Figure 4).

There was significant heterogeneity with I^2^>50% for the outcomes of procedure duration (93%), RF duration (98%), fluoroscopy duration (95%), first-pass isolation (81%), freedom from atrial arrhythmia (73%), and acute PVR (72%). All summary estimates from pooled analyses were made using a random-effects model rather than a fixed-effects model to reduce the influence of heterogeneity between studies. Sensitivity analysis demonstrated the robustness of the above results during the sequential exclusion of studies except for first-pass isolation, freedom from atrial arrhythmia, and acute PVR. Low heterogeneity following exclusion of two studies [28, 31] based on freedom from atrial arrhythmia (I^2^ = 2%) and one study [32] based on acute PVR (I^2^ = 0). Despite reduced heterogeneity, there were no changes in the results of differences between two the groups. Funnel plots and Egger's regression tests of the outcomes of the primary pooled analysis are shown in Supplementary Materials, Figure S1.

### 3.4. Subgroup Analysis

#### 3.4.1. Studies with the Guidance of AI/LSI in Ablation

There were 5 studies [16, 21, 24, 29, 30] with a total of 739 patients (366 in the HPSD group, 373 in the conventional group) that ablated with the guidance of AI or LSI. Whether with the guidance of AI or LSI, total procedure duration (MD −21.08 min (95% CI −24.63 to −17.54); *P* < 0.001) and RF duration [MD −9.43 min (95% CI −12.21 to −6.65); *P* < 0.001] (Supplementary Materials, Figures S2, (a), (b)) were shorter in the HPSD RFA group. Guided by AI/LSI, there was no apparent difference in freedom from atrial arrhythmia at one year (OR 1.41, 95% CI 0.88–2.25, *P*=0.15 and PVR (OR 1.55, 95% CI 0.40–5.98, *P*=0.52) (Supplementary Materials, Figures S2, (c), (d)) between the two groups. However, the HPSD RFA group demonstrated higher freedom from atrial arrhythmia at one year (OR 1.66, 95% CI 1.12–2.47, *P*=0.01) and lower PVR (OR 0.32, 95% CI 0.17–0.61, *P*=0.008) (Supplementary Materials, Figures S2, (c), (d)) without the guidance of AI/LSI.

#### 3.4.2. Studies with 50 W vs 40–50 W in the High-Power Short-Duration Radio Frequency Ablation Group

In the HPSD RFA group, there were 9 studies [16, 17, 23–25, 27–29, 32] where ablation was performed with a setting of 50 W, while 7 studies [18, 19, 21, 22, 26, 30, 31] with a power setting of 40–50 W. To reduce heterogeneity, two studies [20, 32] exceeding the power of 50 W were excluded. Without increased complication rates, freedom from atrial arrhythmia at one year was higher in the HPSD RFA group with the power setting of 40–50 W (*P*=0.03), conversely, no difference was found in this endpoint between the two groups with the power setting of 50 W (*P*=0.52) (Supplementary Materials, Figure S3). At 50 W or 40–50 W, total procedure duration (*P* < 0.001), total RF duration (*P* < 0.001), and fluoroscopy duration (*P* < 0.001) (Supplementary Materials, Figure S4) were both significantly shorter in the HPSD RFA group.

## 4. Discussion

This meta-analysis provides a more comprehensive assessment of HPSD RFA and conventional RFA in patients with AF. As in the previous studies [8, 9, 33, 34], our results suggest that HPSD RFA may be more effective with higher first-pass isolation and freedom from atrial arrhythmia and lower acute PVR when compared with conventional RFA. Additionally, there was no difference in safety outcomes between the two groups. However, our study had more findings. In our study, there was no difference in PVR between the two groups that described redo procedures. In subgroup analysis, there was no difference between the two groups using AI/LSI-guided ablation for freedom from atrial arrhythmia. And HPSD group with a power setting of 40–50 W other than 50 W had better efficacy when compared with the conventional group.

PVI is the cornerstone of AF ablation [35], however, PVR is frequent and is mostly the result of catheter instability, tissue edema, and a reversible nontransmural injury [36]. One of the main reasons for AF recurrence is the recovery of the conduction between the pulmonary veins and left atrium [37], so continuous and transmural lines are key to the success of ablation. In animal studies, the lesions were wider and HPSD ablation resulted in 100% contiguous lines with transmural lesions which improved lesion-to-lesion uniformity [38]. In 6 swine, HPSD ablation was performed using the QDOT MICRO^TM^ Catheter at a setting of 90 W for 4 s and conventional ablation was delivered using a Thermocool Smarttouch SF Catheter at a setting of 30 W for 30 s, Barkagan et al. found that all lines remained intact after 30 days in HPSD ablation, while none of the lines were continuous in conventional ablation [39]. Although there was variation in the definition of freedom from arrhythmia in each study and the use of AADs, our analysis favors the HPSD RFA strategy over the LPLD RFA strategy for lower acute PVR, higher first-pass isolation, and higher freedom from atrial arrhythmia. Nevertheless, in our analysis, there was no difference between the two groups in PVR during the redo procedure. Some patients might have had recurrence during the followup period, but they did not undergo redo procedures. Furthermore, the followup was determined to be one year, therefore all the reasons above may underestimate the rate of chronic PVR.

However, the appropriate power for the RF ablation is not clear. One study [32] used higher power of 70 W for 5–7 s and demonstrated significantly less arrhythmia recurrence during one-year followup (26.9% vs 34.9%, *P* < 0.013) with no major complications. The QDOT-FAST trial [40] used 90 W for 4 s per site in 52 patients with paroxysmal atrial fibrillation and 94.2% of patients were in sinus rhythm at 3 months with one pseudoaneurysm and one asymptomatic thromboembolism. In our meta-analysis, mostly half of the studies of HPSD RFA used 50 W, and the others used 45–50 W. For freedom from atrial arrhythmia at one year, the HPSD RFA group demonstrated higher efficacy with the power setting of 45–50 W, whereas the two groups were similar with the power setting of 50 W. To reduce complications when ablating with 50 W on the posterior atrial wall, ablation duration was shorter than that of 40/45 W. Transmural damage may not be achieved because the lesion is shallower，as well as in less total energy, resulting in no difference in the recurrence rate between the two groups [41]. Winkle et al. [42] reported that 6 independent predictors affected the outcomes for HPSD ablation including age, gender, type of AF, left atrial size, type of catheter, and posterior wall isolation. Therefore, further studies will be required to explore the most optimal power and duration for HPSD RFA to bring the highest clinical value.

Previous studies indicate force-time integral (FTI) as a target value to achieve permanent PVI, while not considering power settings. Consequently, only 72% of PVs remained isolated in 3 months [43]. AI is a novel ablation quality marker that incorporates contact force (CF), time, and power in a weighted formula and LSI is a multiparametric index incorporating CF and radiofrequency current data across time. Many reports demonstrated that AI or LSI can be used as the correlation index of pulmonary vein persistent isolation [44, 45]. HPSD group had a lower recurrence of atrial arrhythmia at 12 months, higher first-pass isolation, lower acute PVR, and similar complication rates in the AI-guided group compared with a non AI-guided group [33, 46]. Moreover, HPSD-AI or LSI groups might increase freedom from atrial arrhythmia for patients with additional ablation beyond PVI [33]. Okamatsu et al. [47] studied a group of persistent AF patients undergoing AI-guided PVI with target values of 550 for anterior and 400 for posterior left atrial regions, with 22% patients demonstrating late PVR during repeat procedures after 2 months and 95% patients were in sinus rhythm at 12 months. However, freedom from atrial arrhythmia and acute PVR failed to demonstrate a significant advantage with AI or LSI in our analysis. Regrettably, only 5 studies were included in our subgroup analysis with AI or LSI-guided procedure, of which only 4 studies and 2 studies respectively illustrated freedom from atrial arrhythmia at one year and acute PVR rates, and each study had different ablation strategies, antiarrhythmic drug use, and recurrence of arrhythmia definition which might influence these results. We did not analyze first-pass isolation because only one study reported this data. Therefore, more well-designed and large-scale RCTs are required to confirm these findings.

Safety during elective PVI procedures is of worthwhile concern. Radiofrequency catheter ablation is a technique where conductive and resistive heating are delivered through electrode catheters to myocardial tissue creating a thermal lesion. Irreversible myocardial tissue injury with cellular death occurs once the temperature of approximately 50°C has been reached, whereas conductive heating transfers thermal energy directly to deeper tissue [48]. Unlike conventional ablation, the HPSD ablation strategy results in a higher resistive heating and lower conductive heating, which may reduce collateral injury to surrounding structures such as the esophagus [38, 49]. Late gadolinium enhancement MRI of the esophagus in 574 patients following AF ablation using HPSD settings of 50 W for 5 seconds reported a 14.3% incidence of moderate to severe thermal oesophageal late gadolinium enhancement with no fistulas [23]. Takemoto et al. [50] reported that high-power settings based on the AI or LSI might reduce the collateral thermal damage by comparing the use of 20 W and 40 W with the same AI or LSI for RF applications. In 10284 patients, HPSD RFA strategies performed at 45–50 W have very low complication rates with 1 death due to an atrioesophageal fistula and 33 cases of cardiac tamponade [51]. In our analysis, only one case of cardiac tamponade occurring in the conventional RFA group was reported by Hamsom et al. [25], and cases of aterioesophageal fistula were not observed in each group. Our results demonstrated that the HPSD RFA strategy appears to be as safe as the conventional RFA strategy.

In terms of procedure duration, RF duration, and fluoroscopy time, the HPSD RFA strategy represents distinct advantages compared with the conventional RFA strategy whether in the subgroup analysis or not. Additionally, the reduction in procedure times can decrease the intravenous fluid volumes administered to patients which may benefit patients with cardiac insufficiency. Finally, less radiation exposure will also benefit both patients and physicians [9].

To conclude, our results of the pooled analysis favor the use of HPSD settings over conventional settings. However, more RCT studies are needed to further assess the above results.

### 4.1. Limitations

We acknowledge several limitations in our study. First, we have only one RCT included in our meta-analysis while the rest were nonrandomized comparative studies. Although, all included studies were of good quality based on the Newcastle Ottawa scale, reflecting a real-world experience, more randomized controlled trials would provide better evidence for the difference in outcomes between two groups. Second, there were variations in each study in terms of power, types of catheters, contact force, target temperature, antiarrhythmic drug use, and the definition of freedom from atrial arrhythmia, resulting in significant heterogeneity between groups. And seldom included studies analyzed total energy during ablation procedure which we could not compare between two groups. Third, the included studies not only performed PVI but also additional linear ablation and different surgical methods might affect the maintenance of sinus rhythm. At last, we have a limited number of studies that reported PVR during redo procedures and with the guidance of AI/LSI. Finally, the exact anatomical locations of PVR were not clearly described in each study, so we could not analyze the specific locations of PVR.

## 5. Conclusions

High-power short-duration RFA was related to better procedural effectiveness and higher freedom from atrial arrhythmia with comparable safety when compared with conventional RFA. Additionally, HPSD RFA decreases procedural, RF, and fluoroscopy durations. Meanwhile, in the subgroup analysis, HPSD RFA demonstrates a feasible, effective and safe approach for AF ablation.

## Figures and Tables

**Table 1:** Procedural characteristics.

Study	^+^N (HPSD group vs conventional group)	RFA catheter	System	HPSD RFA strategy	Conventional RFA strategy	Ablation-guided (LSI or FTI or AI)	Ablation strategy	Esophageal temperature monitoring	Follow-up duration (months)	Monitoring methods	Design	Freedom from arrhythmia definition
Nilsson et al. [19]	45 vs. 45	Irrigated	^+^NR	45 W, 55°C, 20 s	30 W, 50°C, 120 s	^+^NR	^§^PVI	^+^NR	15 ± 7	Out-patient clinic visit, telephone interview	Retrospective	Free of ^+^AF. with ^*∗*^AADs

Baher et al. [23]	574 vs. 113	^+^CF (ThermoCool SmartTouch) and nonCF	^+^C	50 W, 10–20 g, 5 s, dragging technique, ^§^ILD 4-5 mm	≤35 W, 10–20 g, 10–30 s, dragging technique, ^§^ILD 4-5 mm	^+^NR	^§^PVI ± lines	Stopped with increasing temperature Of 2°C	30	Home monitoring, ^§^ECG	Retrospective	Free of ^+^AF/^§^AFL/ablation/cardioversion

Pambrun et al. [22]	50 vs. 50	Irrigated, SmartTouch	^+^C	50 W elsewhere, 40 W posterior, ≥10 g, 2 s after ^+^USM, point‐by‐point manner	30 W elsewhere, 25 W posterior, ≥10 g, 2 s, point‐by‐point manner	^+^USM	^§^PVI	^+^NR	12	^§^ECG, Holter	Prospective	Free of any ^+^ATAs without ^*∗*^AADs

Bunch et al. [28]	402 vs. 402	Irrigated, ^+^CF	^+^C	50 W, 5–20 g, 5–15 s elsewhere, 5 s posterior, point‐by‐point manner	30 W, 10–20 s elsewhere, 5 s posterior, point‐by‐point manner	Local electrograms and dwell time.	^§^PVI ± lines	^+^NR	36	Ambulatory monitoring, ^§^ECG, event monitor	Retrospective	Free of ^+^AF/^§^AFL without ^*∗*^AADs

Ejima et al. [17]	60 vs 60	Irrigated, ^+^CF (ThermocoolSmartTouch)	^+^C	50 W, 5–20 g, 3-5 s after ^+^USM, ^§^ILD <5 mm	25–40 W, 10–20 g, 5–10 s after ^+^USM, 8 ml/min, ^§^ILD <5 mm	^+^USM	^§^PVI ± lines	Yes, stopped if the temperature rises to >39°C	Conventional group: 20.7 ± 2.0；HPSD group:12.5 ± 2.9	^§^ECG, Holter	Prospective	Free of any ^+^ATAs without ^*∗*^AADs

Kottmaier et al. [32]	97 vs 100	Flexibility SE catheter	^+^NR	70 W, 7 s elsewhere, posterior wall 5 s, 20 ml/min, point‐by‐point manner	30–40 W, 20–40 s, 17 ml/min, point‐by‐point manner	^+^NR	^§^PVI	Yes, the upper limit temperature of 42°C	12	^§^ECG, Holter, clinical Evaluation	Prospective	Free of any ^+^ATAs without ^*∗*^AADs

Kumagai et al. [24]	80 vs. 80	^+^CF (HPSD RFA) and nonCF (conventional RFA)	^*∗*^E	50 w, 5–10 g, 30 ml/min, ^*∗*^LSI 5; near the esophagus <10 g, 5 s	30–40 w, 30 s, 8–15 ml/min, near the esophagus 20 W	^*∗*^LSI	^§^PVI ± lines	Yes, stopped if the temperature rises to >40°C	12	^§^ECG, Holter	Retrospective	Free of any ^+^ATAs with ^*∗*^AADs

Kyriakopoulou et al. [21]	80 vs. 105	Irrigated, ^+^CF (ThermocoolSmartTouch)	^+^C	40 W, 30 ml/min, ILD <6 mm, posterior wall/roof/south pole ^*∗*^AI ≥400, anterior≥550, point‐by‐point technique	30 W, 30 ml/min, ILD <6 mm, posterior wall/roof/south pole AI ≥400, anterior≥550, point‐by‐point technique	^*∗*^AI	^§^PVI ± lines	Yes, stopped if the temperature rises to >38.5°C	12	Holter	Retrospective	Free of any ^+^ATAs without ^*∗*^AADs

Yavin et al. [26]	112 vs 112	Irrigated, ^+^CF (ThermocoolSmartTouch)	^+^C	45–50 W, 15 s, 8 s Posterior, 17 ml/min, point‐by‐point technique	30–40 W, 30 s elsewhere; posterior wall 20 W, 20 s, 8–17 ml/min, point‐by‐point technique	^+^NR	^§^PVI ± lines	Yes, stopped if the temperature rises to >39°C	HPSD group: 14.4; Conventional group: 22.8	Holter	Prospective	Free of any ^+^ATAs

Yazaki et al. [27]	32 vs. 32	Irrigated (ThermoCool STSF)	^+^C	50 W, 5–15 g, 5–10 s after ^+^USM, ILD＜6 mm	25–40 W, near the esophagus 20–25 W, 10–20 g, ILD＜6 mm，	^+^USM	^§^PVI ± lines	Yes, the upper limit temperature of 42°C	12	Portable electrocardiographic monitoring, patient symptoms, ^§^ECG	Retrospective	Free of any ^+^ATAs without ^*∗*^AADs

Chen et al. [18]	40 vs 40	Irrigated, ^+^CF	^*∗*^E	40–50 W, 10 s, ≥10 g	25–30 W, 17–30 ml/min, ^+^FTI = 400 gs	^+^FTI	^§^PVI	^+^NR	12	Holter	Retrospective	Free of ^+^AF with ^*∗*^AADs

Dikdan et al. [16]	76 vs. 51	Irrigated, ^+^CF	^*∗*^E	50 W, 8–40 g, 15 s, 30 ml/min, anterior ^*∗*^LSI 6, posterior ^*∗*^LSI 5	25–40 W, 10–40 g, 30–60 s, 17 ml/min, LSI 4.5–5.5	^*∗*^LSI	^§^PVI ± crina	Yes, stopped if the temperature rose by 0.2°C or more	12	Mobile cardiac outpatient telemetry monitors if patients have symptoms, ^§^ECG	Retrospective	Free of ^+^AF with ^*∗*^AADs

Hansom et al. [25]	107 vs. 107	Irrigated, ^+^CF	^+^C	50 W, 10–20 g, 8–10 s elsewhere, 6-8 s posterior, point‐by‐point manner, ^§^ILD＜4 mm	^+^FTI≥400 g·s，30 W, 10–20 g, 30–40 s, posterior, 300≤FTI≤400 g·s, 25 W, 20–25 s, point‐by‐point manner, ^§^ILD＜4 mm	^+^FTI	^§^PVI ± lines	Yes	12	^§^ECG, outpatient clinical assessments.	Prospective	Free of any ^+^ATAs at one year with ^*∗*^AADs

O'Brien et al. [29]	88 vs. 93	^+^CF (ThermoCoolSmartTouch)	^+^C	50 W, anterior wall 40 g, ^*∗*^AI 550, 30 ml/min, ^§^ILD＜5 mm, posterior wall 30 g, ^*∗*^AI 400, 17 ml/min, ^§^ILD＜6 mm, point‐by‐point manner	35–40 W, ^§^ILD 6 mm, point‐by‐point manner	^*∗*^AI	^§^PVI ± lines	Yes, stopped if the temperature rise >38°C	12	^§^ECG, Fibricheck mobile Phone	Retrospective	Free of any ^+^ATAs with ^*∗*^AADs

Park et al. [20]	315 vs. 945	Irrigated；only 117 patients in the conventional group used ^+^CF catheters	^+^C	242 patients: 50 W, 10–15 s, posterior wall 40 W, ＜10 s; 73 patients: anterior wall 60 W, posterior wall 50 W	30–35 W anterior, 20–25 W posterior; When used ^+^CF	^*∗*^AI	^§^PVI ± lines	Yes, stopped if the temperature rise to >38.4°C	12	^§^ECG, Holter	Retrospective	Free of ^+^AF without ^*∗*^AADs

Vassallo et al. [31]	197 vs. 158	^+^CF (TactiCat™ contact force sensing catheter)	^*∗*^E	50 W anterior, 5–10 g, 45 W posterior, 10–20 g, 6 s, 35 ml/min, dragging technique	30 W anterior, 20 W posterior, 10–20 g, 30 s, 17 ml/min, dragging technique	^+^NR	^§^PVI	Yes, stopped with an increasing temperature Of 1°C	^+^HPSD group: 22.35; Conventional group: 28.45	^*∗*^ECG, Holter	Prospective	Free of ^+^AF

Wielandts et al. [30]	48 vs. 48	^+^CF (ThermoCoolSmartTouch)	^+^C	45 W, ≤30 g, anterior wall AI>550, posterior wall ^*∗*^AI>400, 30 ml/min, ^+^ILD≤6 mm, point‐by‐point manner	35 W, 30 ml/min, ^+^ILD≤6 mm, point‐by‐point manner	^*∗*^AI	^§^PVI ± lines	Yes, stopped if the temperature rise to >38.5°C	6	^+^ECG, Holter	Prospective and ^*∗*^RCT	Free of ^+^AF with ^*∗*^AADs

^*∗*^AI: ablation index; ^+^ATAs: atrial tachyarrhythmias; ^+^AF: atrial fibrillation; ^§^AFL: atrial flutter; ^*∗*^AADs: antiarrhythmic drugs; ^+^CF: contact force; ^+^C : CARTO; ^§^ECG: electrocardiograph; ^*∗*^E : Ensite; ^+^FTI: force-time integral; ^+^HPSD: high-power short-duration; ^§^ILD: interlesion distance; ^*∗*^LSI: lesion index; ^+^N: number of patients; ^+^NR: no records; ^§^PVI: pulmonary vein isolation; ^*∗*^RCT: randomized controlled trial; ^+^USM: unipolar signal modification.

**Table 2:** Baseline characteristics.

Study	^*∗*^ *G*	*N*	Paroxysmal AF(%)	Mean age (years)	Male (%)	Hypertension (%)	Diabetes (%)	Previous Stroke/TIA (*n*,%)	CHA2DS2‐VASc score, median	LA volume /LA size	LVEF(%)	Recurrence during the follow-up period(%)	complications
Nilsson et al. [19]	HPSD +RFA	45	26 (57%)	55 ± 10	30 (66.7)	21	+NR	NR	NR	NR	NR	11 (24.4)	1 (one experienced transient cerebral ischaemic episode)
	Conventional +RFA	45	32 (71%)	51 ± 11	36 (80)	20	NR	NR	NR	NR	NR	12 (26.7)	1 (one experienced transient cerebral ischaemic episode)

Baher et al. [23]	HPSD +RFA	574	276 (46.8)	69.0 ± 11.8	385 (67.1)	369 (64.2)	112 (19.5)	81 (14.1)	2.9 ± 1.7	NR	NR	241 (42.0)	82 (all had esophageal thermal injury)
	Conventional +RFA	113	80 (70.8)	68.3 ± 11.6	67 (59.3)	68(60.1)	18(18.5)	7 (6.2)	2.5 ± 1.6	NR	NR	46 (40.7)	16 (all had esophageal thermal injury)

Pambrun et al. [22]	HPSD +RFA	50	50 (100)	65 ± 8.2	35 (70)	14 (28)	3 (6)	3 (6)	NR	107.6 ± 23.1 ml	61.7 ± 5.6	5 (10.0)	2 (all had Groin hematoma)
	Conventional +RFA	50	50 (100)	62.5 ± 10.6	30 (60)	12 (24)	3 (6)	3 (6)	NR	102.9 ± 20.1 ml	61.1 ± 4.4	6 (12.0)	3 (all had Groin hematoma)

Bunch et al. [28]	HPSD +RFA	402	190 (47.3)	67.1 ± 10.5	253(62.9)	358 (89.1)	126(31.1)	47 (11.7)	NR	NR	54.6 ± 12.1	15 5(38.6)	45 (Thirty-three patients dead and twelve patients with stroke)
	Conventional +RFA	402	202 (50.2)	66.4 ± 12.2	262(65.2)	348 (86.6)	121(30.1)	51 (12.7)	NR	NR	54.7 ± 12.8	135(33.6)	40 (Thirty-one patients dead and nine patients with stroke)

Ejima et al. [17]	HPSD +RFA	60	60 (100)	63.0 ± 11.3	44 (73.3)	29 (48.3)	10 (16.7)	6 (10.0)	1.8	34.3 ml/m2	57.7 ± 3.9	7 (11.7)	1 (all had phrenic nerve palsy)
	Conventional +RFA	60	60 (100)	66.7 ± 8.9	42 (70.0)	30 (50.0)	12 (20.0)	7 (11.7)	2.2	36.1 ml/m2	57.4 ± 6.3	17 (28.3)	0

Kottmaier et al. [32]	HPSD +RFA	97	97 (100)	60.8 ± 13.9	57 (58.8)	56 (57.7)	NR	6 (6.2)	1.95	NR	57 ± 5	16 (16.5)	13 (Three patients with pericardial effusion and ten patients had groin complications)
	Conventional +RFA	100	100 (100)	60.8 ± 10.5	60 (60.0)	58 (58.0)	NR	7 (7.0)	1.64	NR	55 ± 9	35 (35.0)	17 (two patients with pericardial effusion and five patients with groin complications)

Kumagai et al.[24]	HPSD +RFA	80	20 (25)	63.0 ± 9.1	60 (75)	NR	NR	NR	0.7 ± 1.0	41.6 ± 5.1 mm	62.5 ± 7.7	11 (13.8)	0
	Conventional +RFA	80	24 (30)	63.1 ± 9.1	66 (82.5)	NR	NR	NR	0.8 ± 0.8	43.3 ± 6.4 mm	62.2 ± 7.2	19 (23.8)	0

Kyriakopoulou et al.[21]	HPSD +RFA	80	80 (100)	67	47 (59)	NR	NR	NR	2	43 ± 8 mm	NR	20 (25.0)	0
	Conventional +RFA	105	105 (100)	64	65 (62)	NR	NR	NR	2	44 ± 6 mm	NR	14 (13.3)	0

Yavin et al. [26]	HPSD +RFA	112	76 (67.8)	62.3 ± 5.2	71 (63.3)	70 (62.5)	11 (9.8)	NR	2.4 ± 1.3	44.2 ± 4.7 mm	60.3 ± 6.1 (n = 91)	23 (20.5)	1 (One patient with steam pop)
	Conventional +RFA	112	67 (59.8)	64.8 ± 7.2	79 (70.5)	76 (67.8)	7 (6.2)	NR	2.6 ± 1.4	47.1 ± 5.1 mm	57.8 ± 5.4 (n = 86)	34 (30.4)	2 (One patient with large pericardial effusion and one patient with right phrenic nerve paralysis)

Yazaki et al. [27]	HPSD +RFA	32	22 (89)	61 ± 11	27 (84)	NR	NR	NR	NR	40 ± 13 ml/cm2	55 ± 7	9 (28.1)	1 (One patient with acute right phrenic nerve injury)
	Conventional +RFA	32	20 (63)	66 ± 11	20 (63)	NR	NR	NR	NR	41 ± 14 ml/cm2	56 ± 7	11 (34.4)	0

Dikdan et al. [16]	HPSD +RFA	76	32 (42.1)	63.20	54 (71.1)	NR	NR	NR	2	145.4 ml (By CTA)	56.8	15 (19.7)	NR
	Conventional +RFA	51	21 (41.2)	60.70	40 (78.4)	NR	NR	NR	1	162.3 m (By CTA) l	56.6	13 (25.5)	NR

Chen et al. [18]	HPSD +RFA	40	30 (75)	56.9 ± 10.6	26 (65.0)	10 (25.0)	2 (5.0)	0 (0)	2.1 ± 1.10	36.4 ± 4.30 mm	64.2 ± 6.9	7 (17.5)	2 (two patients with steam pop)
	Conventional +RFA	40	25 (62.5)	56.70 ± 11.5	29 (72.5)	8 (20.0)	3 (7.5)	0 (0)	2.2 ± 1.00	36.4 ± 4.30 mm	66.4 ± 5.3	13 (32.5)	0

Hansom et al. [25]	HPSD +RFA	107	67 (65)	62 ± 9	69 (65)	44 (41)	9 (8)	4 (4)	1.9	41 ± 0.7 mm	NR	23 (21.5)	3 (One patient with vascular access issues, one patient with urosepsis and one patient with phrenic nerve palsy)
	Conventional +RFA	107	60 (56)	62 ± 9	81 (76)	47 (44)	13 (12)	9 (8)	2.0	41 ± 0.6 mm	NR	29 (27.1)	6 (two patients with vascular access issues, one patient with pericardial tamponade, two patients with urosepsis and one patient with gastroparesis)

O'Brien et al. [29]	HPSD +RFA	88	39 (44.3)	64.5	57 (64.8)	35 (39.8)	6 (6.8)	9 (10.2)	2.0	40 mm	NR	17 (19.3)	6 (One patient with pulmonary edema, three patients with chest pain, and two patients with femoral access site bleeds)
	Conventional +RFA	93	60 (64.5)	64	64 (68.8)	37 (39.8)	8 (8.6)	8 (8.6)	1.0	39 mm	NR	16 (17.2)	2 (One patient with chest pain, and one patient with transient dysphagia)

Park et al. [20]	HPSD +RFA	315	180 (57.1)	59 ± 11	232 (73.7)	151 (47.9)	53 (16.8)	38 (12.1)	1.8 ± 1.6	42.8 ± 6.6 mm	63.2 ± 8.2	41 (13.0)	9
	Conventional +RFA	945	553 (58.5)	59 ± 10	699 (74.0)	455 (48.1)	153 (16.2)	115 (12.2)	1.8 ± 1.5	42.5 ± 6.2 mm	61.5 ± 9.6	171 (18.1)	35

Vassallo et al. [31]	HPSD +RFA	197	113 (66.5)	61.72	141 (71.6)	131 (66.5)	38 (19.3)	14 (7.1)	2.35	NR	NR	32 (16.2)	0
	Conventional +RFA	158	121 (76.58)	58.9	113 (71.5)	105 (66.5)	32 (20.3)	11 (7.0)	2.03	NR	NR	64 (40.4)	0

Wielandts et al. [30]	HPSD +RFA	48	48 (100)	64 ± 11	32 (66.7)	NR	NR	NR	1	39 ± 7 mm	NR	5 (10.4)	1 (all with esophageal lesions)
	Conventional RFA	48	48 (100)	61 ± 11	33 (68.8)	NR	NR	NR	1	40 ± 7 mm	NR	4 (8.3)	1 (all with esophageal lesions)

^*∗*^G: group; ^+^NR: no records; ^+^RFA: radiofrequency ablation.

**Table 3:** Newcastle–Ottawa scale scores and quality assessment of included studies.

References	Selection				Comparability	Outcome			Total
	Representativeness	Selection	Ascertainment	Outcome		Assessment	Follow-up	Adequacy	
Nilsson et al. [19]	^*∗*^	^*∗*^	^*∗*^	^*∗*^	^*∗*^	^*∗*^	^*∗*^	^*∗*^	8
Baher et al. [23]	^*∗*^	^*∗*^	^*∗*^	^*∗*^	^*∗*^	^*∗*^	^*∗*^	^*∗*^	8
Pambrun et al. [22]	^*∗*^	^*∗*^	^*∗*^	^*∗*^	^*∗*^	^*∗*^	^*∗*^	^*∗*^	8
Bunch et al. [28]	^*∗*^	^*∗*^	^*∗*^	^*∗*^	^*∗*^	^*∗*^	^*∗*^	^*∗*^	8
Ejima et al. [17]	^*∗*^	^*∗*^	^*∗*^	^*∗*^	^*∗*^^*∗*^	^*∗*^	^*∗*^	^*∗*^	9
Kottmaier et al. [32]	^*∗*^	^*∗*^	^*∗*^	^*∗*^	^*∗*^^*∗*^	^*∗*^	^*∗*^	^*∗*^	9
Kumagai et al. [24]	^*∗*^	^*∗*^	^*∗*^	^*∗*^	^*∗*^^*∗*^	^*∗*^	^*∗*^	^*∗*^	9
Kyriakopoulou et al. [21]	^*∗*^	^*∗*^	^*∗*^	^*∗*^	^*∗*^	^*∗*^	^*∗*^	^*∗*^	8
Yavin et al. [26]	^*∗*^	^*∗*^	^*∗*^	^*∗*^	^*∗*^^*∗*^	^*∗*^	^*∗*^	^*∗*^	9
Yazaki et al. [27]	^*∗*^	^*∗*^	^*∗*^	^*∗*^	^*∗*^	^*∗*^	^*∗*^	^*∗*^	8
Chen et al. [16,18]	^*∗*^	^*∗*^	^*∗*^	^*∗*^	^*∗*^^*∗*^	^*∗*^	^*∗*^	^*∗*^	9
Dikdan et al. [16]	^*∗*^	^*∗*^	^*∗*^	^*∗*^	^*∗*^	^*∗*^	^*∗*^	^*∗*^	8
Hansom et al. [25]	^*∗*^	^*∗*^	^*∗*^	^*∗*^	^*∗*^^*∗*^	^*∗*^	^*∗*^	^*∗*^	9
O'Brien et al. [29]	^*∗*^	^*∗*^	^*∗*^	^*∗*^	^*∗*^	^*∗*^	^*∗*^	^*∗*^	8
Park et al. [20]	^*∗*^	^*∗*^	^*∗*^	^*∗*^	^*∗*^	^*∗*^	^*∗*^	^*∗*^	8
Vassallo et al. [31]	^*∗*^	^*∗*^	^*∗*^	^*∗*^	^*∗*^	^*∗*^	^*∗*^	^*∗*^	8
Wielandts et al. [30]	^*∗*^	^*∗*^	^*∗*^	^*∗*^	^*∗*^^*∗*^	^*∗*^	^*∗*^	^*∗*^	9

^*∗*^ stands for 1 score.

## Data Availability

The data used to support the findings of this study are included within the article.

## Supplementary Materials

Supplementary Materials (1) Supplementary file 1: funnel plots for various outcomes from the primary pooled analysis comparing HPSD RFA with conventional RFA. (2) Supplementary file 2: forest plots for the subgroup analysis of HPSD RFA compared to conventional RFA. (3) PRISMA 2009 Checklist.
