# Supplementary material for: Comparison of Effectiveness and Safety between High-Power Short-Duration Ablation and Conventional Ablation for Atrial Fibrillation: A Systematic Review and Meta-Analysis
Source: J Interv Cardiol. 2022 Aug 16;2022:6013474. doi: 10.1155/2022/6013474 (PMC9398879; doi:10.1155/2022/6013474)

Figure S1 Funnels plots for various outcomes from the primary pooled analysis comparing HPSD RFA with conventional RFA.

1. Total procedure duration (Egger’s test, P=0.06)


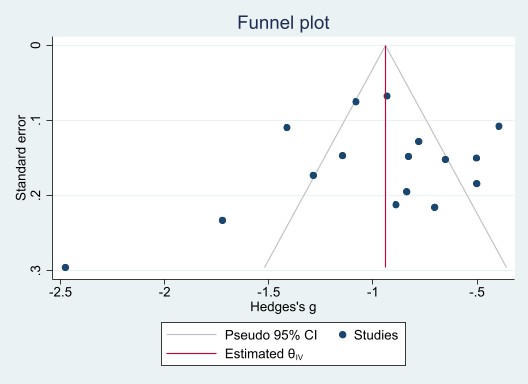


1. Total RF duration (Egger’s test, P=0.049)


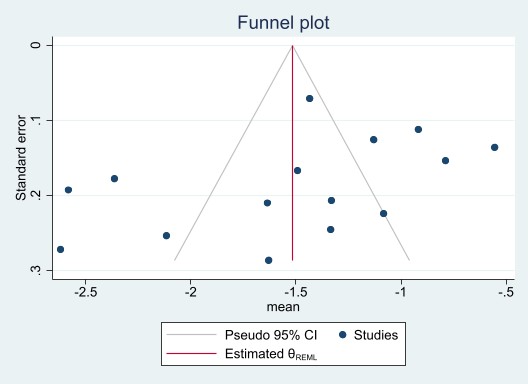


1. Total fluoroscopy duration (Egger’s test, P=0.13)


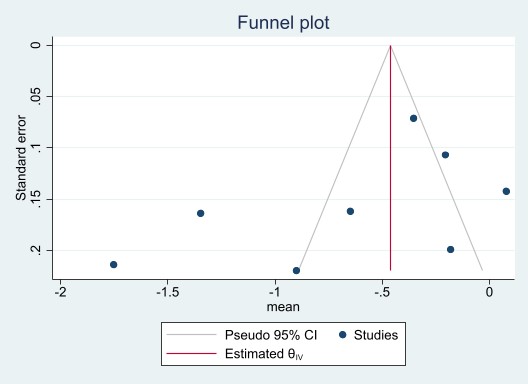


1. First-pass isolation (Egger’s test, P=0.43)


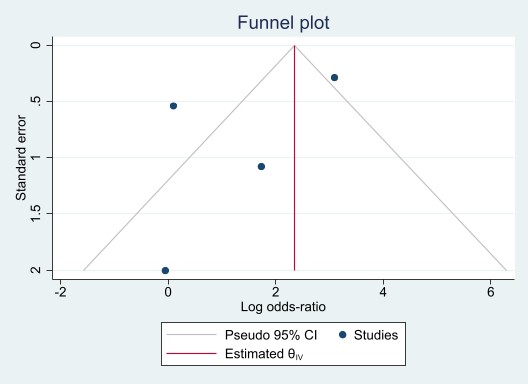


1. Freedom from atrial arrhythmia at one year (Egger’s test, P=0.86)


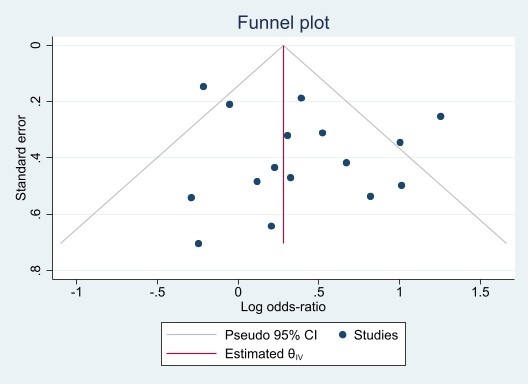


1. Acute PVR (Egger’s test, P=0.75)


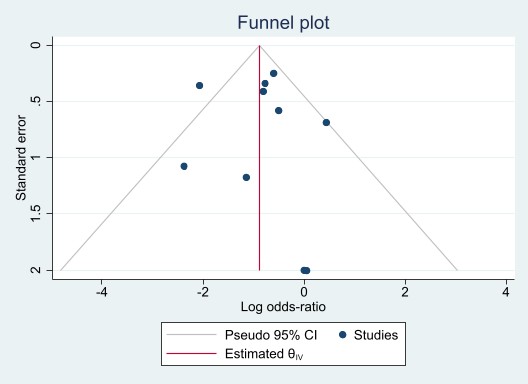


1. Total complications (Egger’s test, P=0.79)


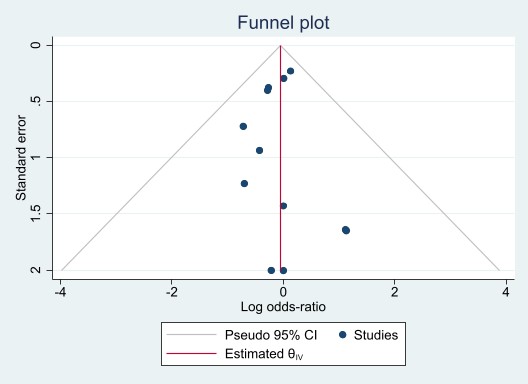


1. PVR during redo procedures (Egger’s test, P=0.18)


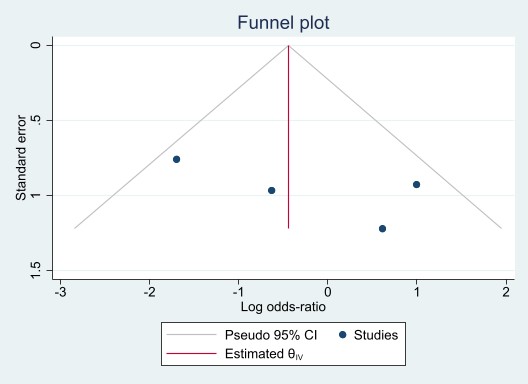

Supplement: Supplementary Materials — (1) Supplementary file 1: funnel plots for various outcomes from the primary pooled analysis comparing HPSD RFA with conventional RFA. (2) Supplementary file 2: forest plots for the subgroup analysis of HPSD RFA compared to conventional RFA. (3) PRISMA 2009 Checklist. [file 6013474.f1.zip › Supplementary files 1 (1).docx]
