# Supplementary material for: Comparison of Effectiveness and Safety between High-Power Short-Duration Ablation and Conventional Ablation for Atrial Fibrillation: A Systematic Review and Meta-Analysis
Source: J Interv Cardiol. 2022 Aug 16;2022:6013474. doi: 10.1155/2022/6013474 (PMC9398879; doi:10.1155/2022/6013474)

Figure S2

1. Total procedure duration in the subgroup analysis with the guidance of AI/LSI in ablation


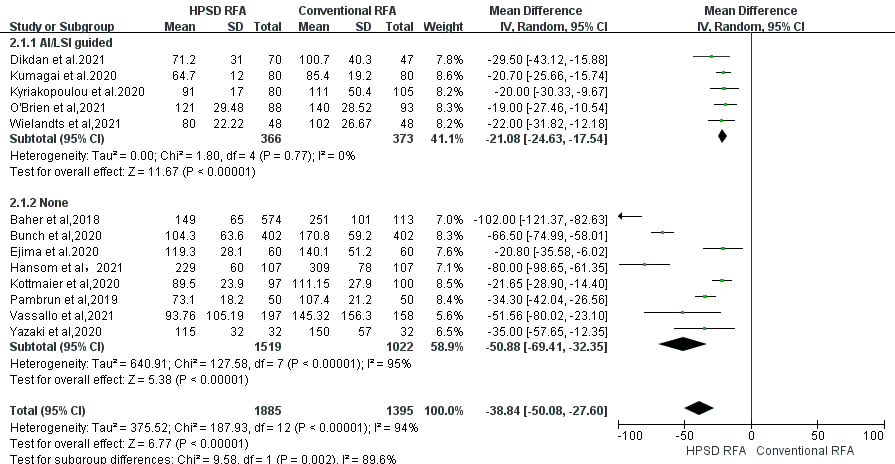


1. RF duration in the subgroup analysis with the guidance of AI/LSI in ablation


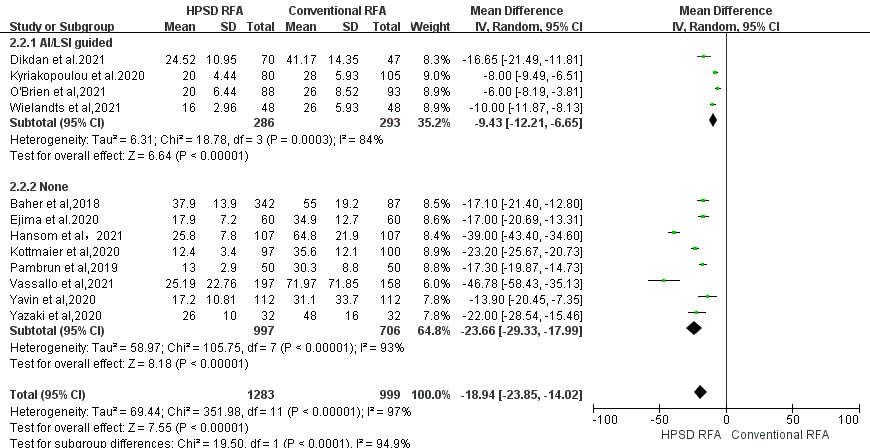


（C）Freedom from atrial arrhythmia at one year in the subgroup analysis with the guidance of AI/LSI in ablation


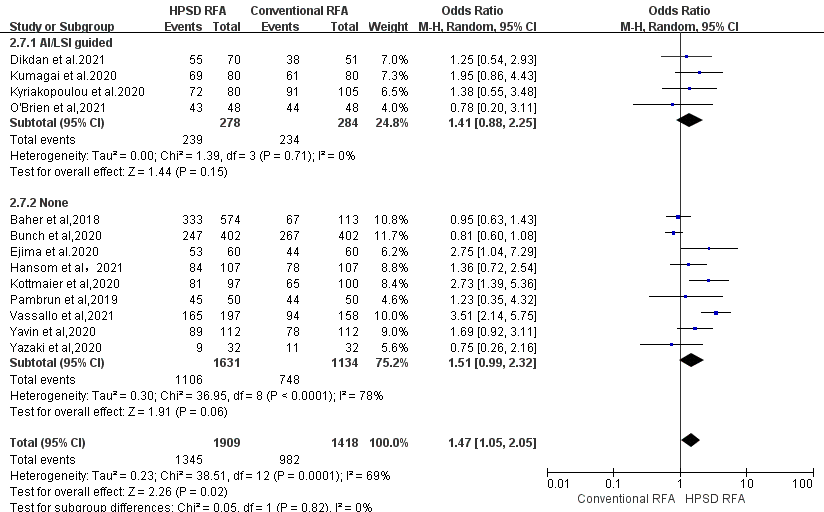


（D）Acute PVR in the subgroup analysis with the guidance of AI/LSI in ablation


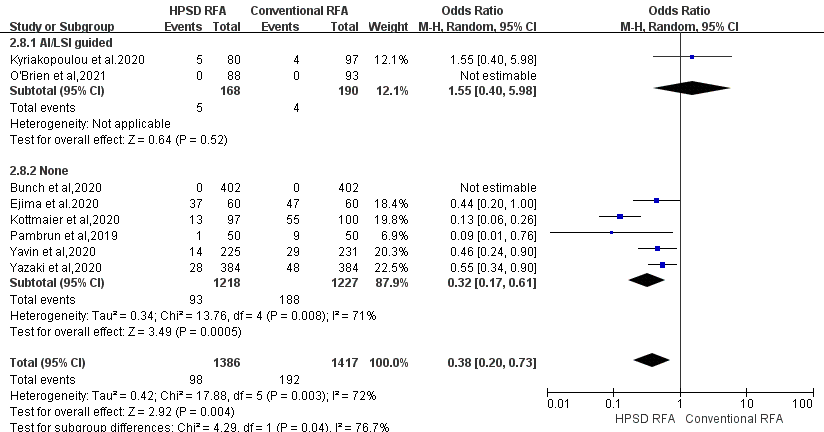


Figure S3

1. Total complications in the subgroup analysis with 50W vs 40-50W in HPSD group


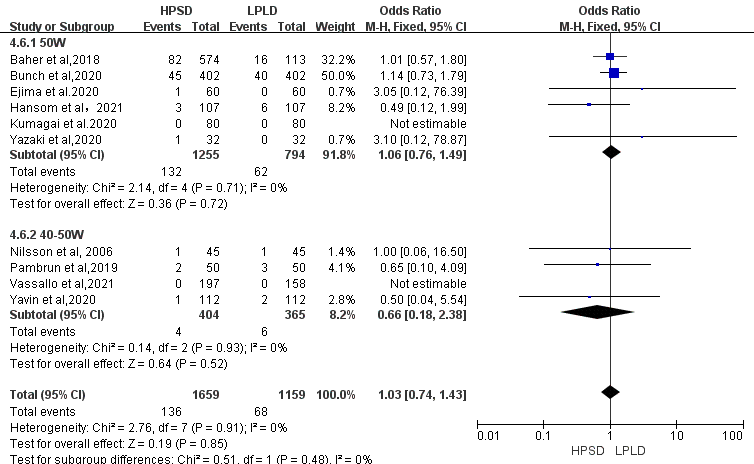


1. Freedom from atrial arrhythmia at one year in the subgroup analysis with 50W vs 40-50W in HPSD group


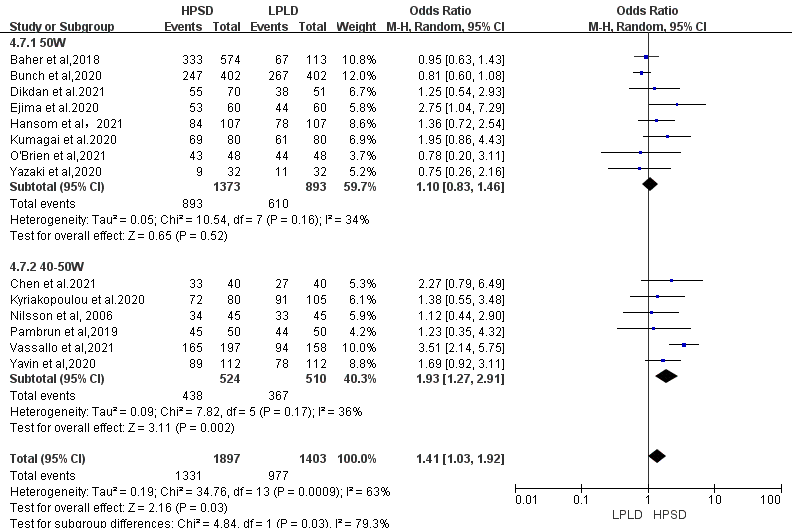


Figure S4

1. Total procedure duration in the subgroup analysis with 50W vs 40-50W in HPSD group


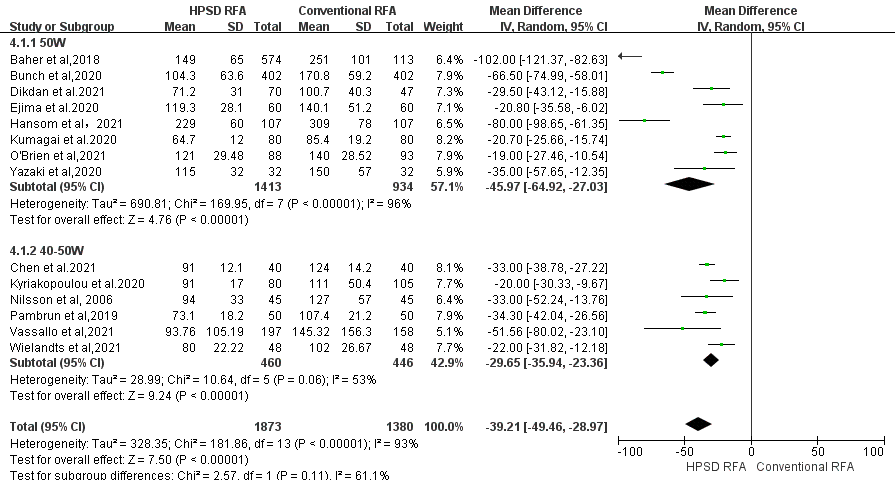


1. RF duration in the subgroup analysis with 50W vs 40-50W in HPSD group


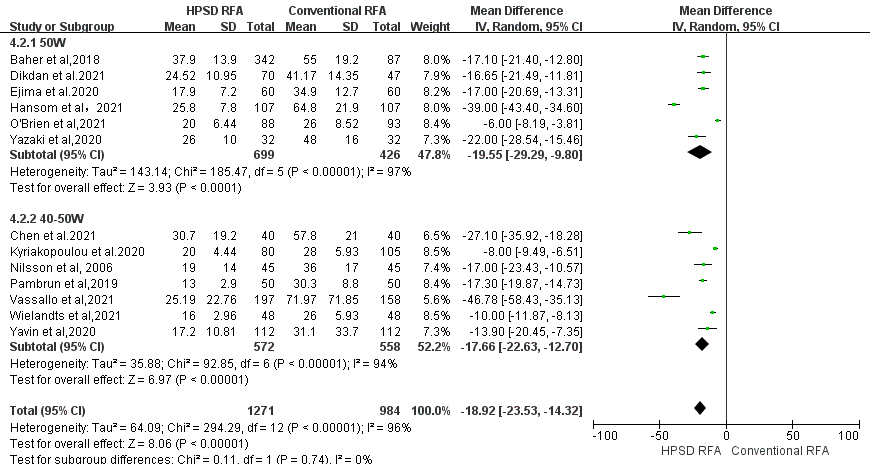


1. Fluoroscopy duration in the subgroup analysis with 50W vs 40-50W in HPSD group


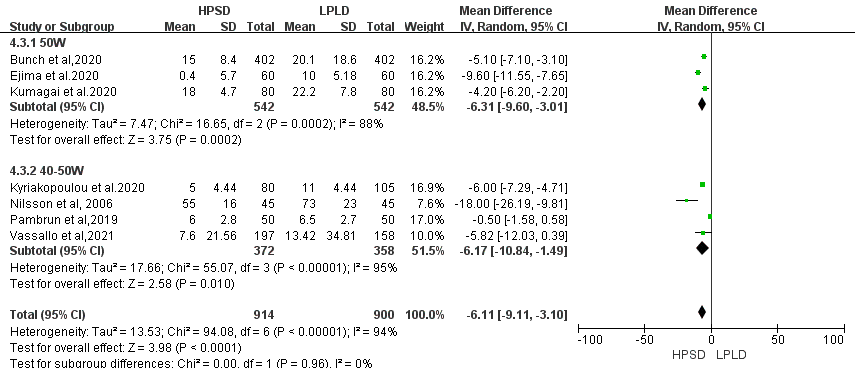

Supplement: Supplementary Materials — (1) Supplementary file 1: funnel plots for various outcomes from the primary pooled analysis comparing HPSD RFA with conventional RFA. (2) Supplementary file 2: forest plots for the subgroup analysis of HPSD RFA compared to conventional RFA. (3) PRISMA 2009 Checklist. [file 6013474.f1.zip › supplementary files 2.docx]
